# Is It Wise to Forget Exercise Stress Echocardiography in the Study of Chest Pain in Children? Comment on Huang, S.-W.; Liu, Y.-K. Pediatric Chest Pain: A Review of Diagnostic Tools in the Pediatric Emergency Department. *Diagnostics* 2024, *14*, 526

**DOI:** 10.3390/diagnostics15091106

**Published:** 2025-04-27

**Authors:** Nuno Cotrim, Carlos Cotrim

**Affiliations:** 1 Cardiology Department do Hospital de Santarém, 2005-177 Santarém, Portugal; 23198@hds.min-saude.pt; 2 Heart Center do Hospital da Cruz Vermelha, 1549-008 Lisboa, Portugal

We read with interest the excellent review manuscript from Huang, S.-W. and Liu, Y.-K. [1], which describes that pediatric chest pain is a common chief complaint in the emergency department. Not surprisingly, children with chest pain are usually brought to the emergency department by their parents out of fear of heart disease. However, chest pain in the pediatric population is generally a benign disease. In this review, we have identified musculoskeletal pain as the most prevalent etiology of chest pain in the pediatric population, accounting for 38.7–86.3% of cases, followed by pulmonary (1.8–12.8%), gastrointestinal (0.3–9.3%), psychogenic (5.1–83.6%), and cardiac chest pain (0.3–8.0%). Various diagnostic procedures for cardiac chest pain are commonly used in the emergency department, including electrocardiogram (ECG), chest radiography, cardiac troponin examination, and echocardiography. However, these examinations demonstrate limited sensitivity in identifying cardiac etiologies, with sensitivities ranging from 0 to 17.8% for ECG and 11.0 to 17.2% for chest radiography. To avoid the overuse of these diagnostic tools, a well-designed standardized algorithm for pediatric chest pain could decrease unnecessary examination without missing severe diseases [2,3,4]. Our primary concern is that no attention has been given to exercise-induced intraventricular gradients, which are easily detectable using exercise stress echocardiography and have been associated with chest pain and other symptoms [5,6,7,8,9,10,11,12,13], including in children. We present the case of a 15-year-old boy, a rugby player, who experienced severe chest pain followed by syncope during a match. Upon evaluation at the emergency department, he showed a significant increase in troponin levels. Coronary angiography (Figure 1) and CT angiography (Figure 2) revealed normal results. However, an exercise stress echocardiogram identified a significant intraventricular gradient (Figure 3).

This was considered the most likely cause of the clinical event. This test was repeated under bisoprolol therapy. In our experience with 139 athletes [11], 58 (41%) were under 18 years old—46 of whom were evaluated for exercise-related symptoms—and 20 (34%) developed an intraventricular gradient during exercise. We strongly advocate for exercise stress echocardiography to be considered for children presenting with exercise-related symptoms in the emergency department at the appropriate time. According to our experience [8], approximately 40% of children with clear exercise-related symptoms, like angina, dizziness, syncope, ST alterations in ECG, or ST alterations in exercise stress ECG (Figure 4), develop mid-ventricular obstruction (MVO) (Figure 5), which appears to be a relatively high prevalence; we recognize this warrants further explanation regarding the mechanisms of development and relationship to chest pain.

In our experience and in the literature [5,6,7,8,9,10,11,12,13,14,15,16], chest pain (exercise angina) has been related to an anatomically small LV chamber, small LVOT, and to an increased relative wall thickness. Additionally, a certain level of hypohydration—characterized by a reduction in left ventricular volumes and commonly linked to intense exercise—may be a potential contributing factor to MVO.As most of the children were referred by other centers, these children were not systematically followed up longitudinally. However, it is our knowledge that four have participated in the genetic study for myocardiopathy and one developed HCM [10]. The increase in intraventricular pressure causing perturbation in subendocardial perfusion is the potential mechanism for ischemia, chest pain, and ST alterations [15,16]. Furthermore, using beta-blockers in children without structural cardiac abnormalities remains a controversial approach. The use of beta-blockers [17,18] is recommended and suitable for pediatric arrhythmias, hypertension, heart failure, hypertrophic cardiomyopathy, migraine prophylaxis, hyperthyroidism, and infantile hemangiomas. Beta-adrenergic receptor antagonists, commonly known as beta-blockers, are divided into three generations based on their receptor selectivity. First-generation beta-blockers (e.g., propranolol) are non-selective and block both β_1_ and β_2_ receptors. Second-generation beta-blockers (e.g., metoprolol) are relatively selective for the β_1_ receptor, while third-generation beta blockers (e.g., carvedilol) block β_1_, β_2_, and α_1_ receptors. Beta-blockers are frequently used to treat adult cardiac conditions, such as hypertension, atrial arrhythmias, and chronic heart failure. Similarly, they are considered a first-line treatment for many pediatric tachyarrhythmias, both in non-operative and peri-operative settings [19]. However, despite their widespread use in children, there is a significant lack of pediatric-specific data to determine precise dosing and personalized treatment. As a result, most pediatric treatment decisions are based on data extrapolated from adult studies. The most commonly prescribed oral beta-blockers for children include atenolol, carvedilol, metoprolol, propranolol, and bisoprolol [19]. The use of beta-blockers is recommended for adult patients with exercise-induced IVPG, whether or not they have hypertrophic cardiomyopathy [20,21,22,23,24]. Based on both our findings and the existing literature, we suggest that children would also benefit from the same treatment (Figure 3).

## Figures and Tables

**Figure 1:**
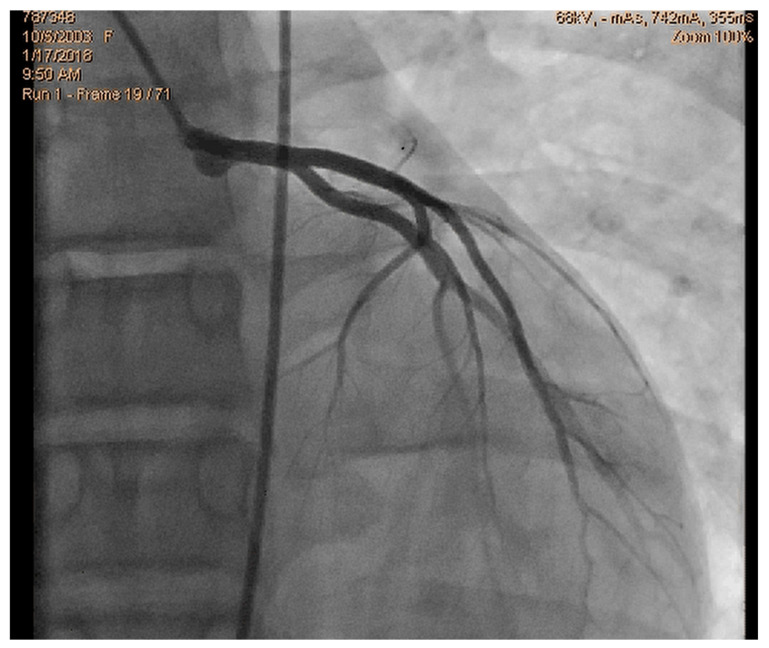
Normal coronary angiography.

**Figure 2:**
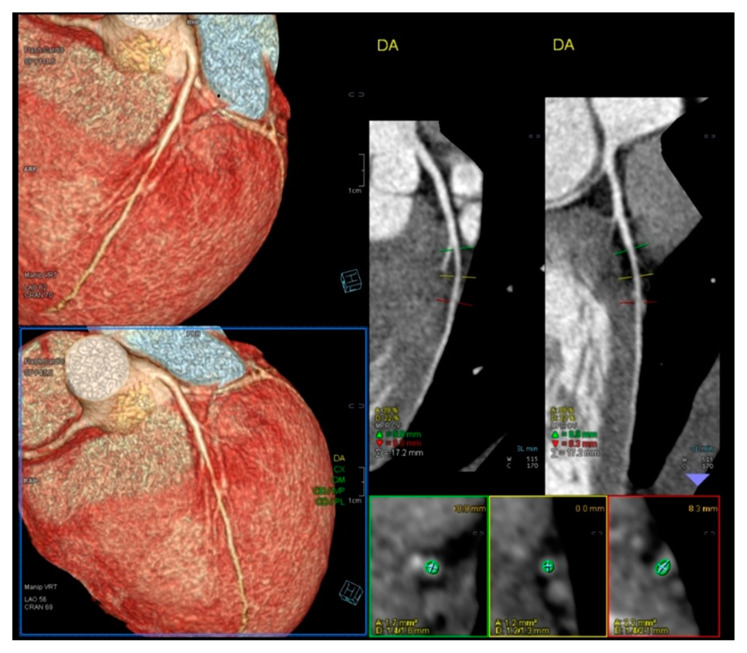
Normal Angio TC of coronary arteries.

**Figure 3:**
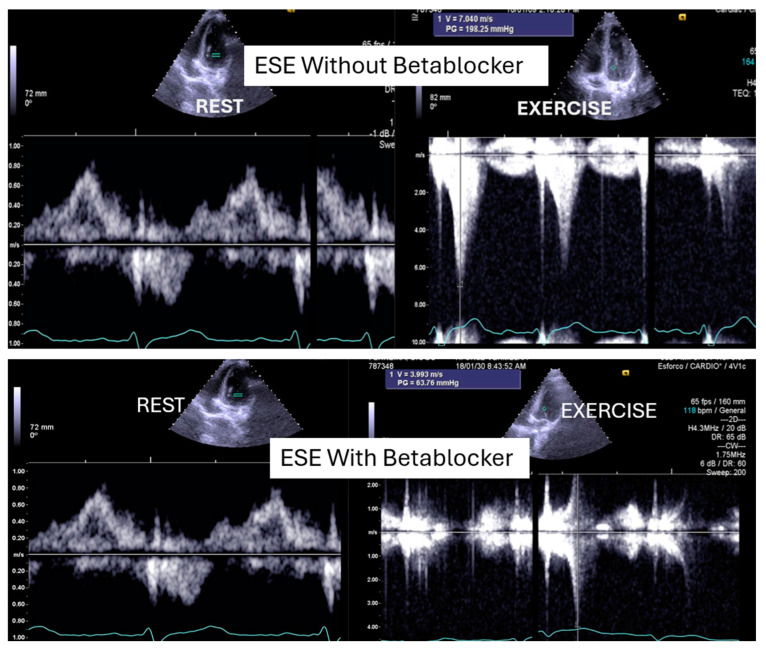
An intraventricular-induced gradient in one child with chest pain followed by syncope during a rugby match. In the upper image, there is a huge intraventricular gradient and in the lower image, there is a small intraventricular gradient under treatment with bisoprolol.

**Figure 4:**
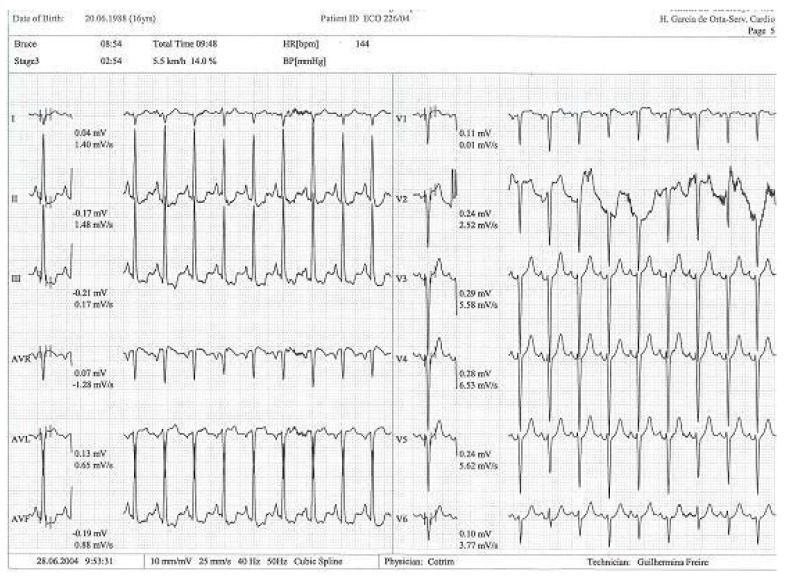
Exercise test with alteration in ST segment in DII, DIII, and avF [14].

**Figure 5:**
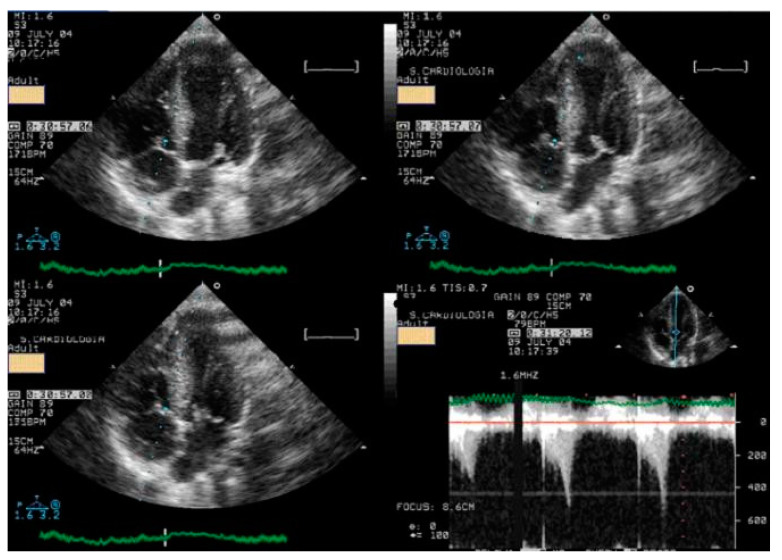
At peak exercise, systolic anterior movement of mitral valve and significant intraventricular gradient was detected [14].
